# Special Issue “Diagnosis, Treatment, and Management of COPD and Asthma”

**DOI:** 10.3390/diagnostics13162634

**Published:** 2023-08-09

**Authors:** Koichi Nishimura

**Affiliations:** 1 Visiting Researcher, National Center for Geriatrics and Gerontology, 7-430, Morioka-cho, Obu 474-8511, Japan; koichinishimura1@gmail.com; 2 Clinic Nishimura, 4-3 Kohigashi, Kuri-cho, Ayabe 623-0222, Japan

It has been my great pleasure to publish 17 papers in the Special Issue “Diagnosis, Treatment, and Management of COPD and Asthma”. I would like to sincerely thank all the contributing authors for submitting their manuscripts. I am proud to have served as Guest Editor for this Special Issue.

On 15 July 2020, I was suddenly offered the job of Guest Editor. I have had more than 30 years of experience in the practice of COPD and asthma since the early 1990s, but since I work in a corner of Asia, I was unsure if I would be able to serve as the Guest Editor of a publication from across the globe. Japan tends to treat respiratory medicine as a unique discipline with a reclusive tendency, and all my life I have been plagued by the closed nature of the field in Japan [1]. My opinion that globalization should be promoted has often been ignored. I have begun this work in the hope that accepting the position of Guest Editor will lead to further exploration of this path.

In Japan, the concept of ACO (Asthma–COPD Overlap) is widespread. For example, when discussing the treatment of stable COPD, it is assumed that inhaled corticosteroids (ICS) should be administered to treat ACO and not for COPD. In other words, in Western countries COPD and asthma are often discussed as two diseases and as one disease group, while in Japan there is a tendency to classify this disease group as three separate diseases: COPD, ACO and asthma. This is one reason why a pro–con discussion on ACO was organized here. Historically, there have been only two pro–con debates regarding whether ICS should be given in COPD, published in the *American Journal of Respiratory and Critical Care Medicine* in 2000 [2,3] and the *European Respiratory Journal* in 2009 [4,5]. Although not identical, the third debate was published based on more than a decade of knowledge on similar issues. We believe that it is possible to produce a paper of great interest to readers. I express my deepest gratitude to the two groups of authors, Peter Calverley and Paul Walker from Liverpool, UK [6] and Naoya Fujino and Hisatoshi Sugiura from Sendai, Japan [7], who gave us the opportunity to publish pro–con reviews in this Special Issue.

Between August 2020 and September 2021, a total of 17 papers were published, comprising 10 original articles and 7 reviews: 12 addressing COPD only, 1 addressing asthma alone, and 4 that addressed both diseases. It has been a pleasure to help facilitate this issue, and I hope that readers will find the articles interesting and informative. I am delighted to have had the opportunity to devote some of my time to writing and to have submitted two original papers [8,9]. As a Guest Editor, I issued a multifaceted call for manuscripts to attract submissions. Some manuscripts were also submitted in response to the call but were not accepted after undergoing peer review, and thus were not published. We thank all those who contributed to this Special Issue. I am very grateful to the Managing Editor, Mr. Dennis Zhu, for giving me the opportunity to serve as a Guest Editor and for his continued support and assistance.
